# The oath and accountability: Insights From The 2nd Annual IDF Medical Corps Bioethics Conference during wartime

**DOI:** 10.1186/s13584-026-00755-2

**Published:** 2026-04-30

**Authors:** Avi Shina, Maya Peled Raz, Noa Revivo Tuchner

**Affiliations:** 1 Medical Services Center, Israel Defense Forces Medical Corps, Tel Hashomer, Ramat Gan, Israel; 2 The Faculty of Medicine, The Hebrew University of Jerusalem, Jerusalem, Israel; 3 The School of Public Health, Faculty of welfare and health Sciences, University of Haifa, Haifa, Israel

**Keywords:** Military medicine, Medical ethics, Military medical ethics, Oath, Professional accountability, Values, Code of ethics

## Abstract

The Israel Defense Forces Medical Corps (IDF MC) assumes statutory responsibility for the health of personnel across all military branches and services within the IDF. Since the outbreak of the “Swords of Iron” war in October 2023, the Corps has operated under unprecedented conditions, treating casualties across multiple fronts. The prolonged conflict has posed immense challenges, raising complex ethical dilemmas that demand real-time processing. In July 2025, the IDF MC convened its second annual bioethics conference, “The Oath and Accountability,” gathering approximately 200 military healthcare leaders. The conference utilized a comparative analysis of the Medical Corps Oath vis-à-vis civilian codes to examine wartime realities. Discussions focused on real-life dilemmas presented by field personnel, exploring the profound tension between the dual identities of the “soldier-healer” and the conflict between clinical duty and operational necessity. Drawing on theoretical frameworks of ethical leadership, the conference underscored that maintaining openness to discuss ethical issues is a critical mechanism for resilience. Key themes included the cultivation of professional integrity and the exercise of “ethical voice” within a hierarchy. The conference concluded that ethical engagement transforms the medical officer from a technical provider into a strategic advisor on values in combat, reaffirming that a robust ethical compass is not a peacetime luxury but a vital operational strength and necessity.

## Introduction

The Israel Defense Forces Medical Corps (IDF MC) occupies a unique and complex position within the landscape of military medicine. It holds statutory responsibility for the health of all military personnel across all branches, providing a continuum of care that spans from point-of-injury combat medicine to routine healthcare, mental health services, and rehabilitation [1]. Furthermore, the Corps is not an isolated island; it is deeply embedded within Israel’s national health system, with its personnel actively integrated into civilian academic centers and hospitals.

Since the abrupt outbreak of the “Swords of Iron” war on October 7th, 2023, the IDF MC has operated under unprecedented conditions. Medical personnel have been tasked with treating casualties across multiple active fronts, providing care to released hostages [2, 3], and managing mass casualty events while under fire. The scope, duration, and intensity of the war have posed immense challenges that extend far beyond the clinical domain.

Wartime reality inevitably generates complex ethical dilemmas. These range from the immediate, such as triage under fire and the treatment of enemy combatants, to the systemic, such as balancing the dual loyalties of the physician-soldier. Moreover, the unprecedented duration of the conflict has introduced a critical challenge: the need to debrief and apply lessons learned in real-time to improve care [4–6]. Unlike short-term operations, prolonged war demands immediate adaptation, yet conducting such a learning process amidst ongoing multi-front battles is far from obvious, as introspection under fire must be performed with extreme humility and sensitivity [7]. To navigate this complexity and recognizing that ethical resilience is not merely a moral imperative but a vital element of operational strength [8], the IDF MC leadership initiated a continuous, proactive learning process. This strategy included the reinstatement of the institutional Ethics Committee to provide statutory guidance to decision-makers and the integration of ethical learning into real-time operational debriefing [9, 10].

As part of this broader strategy, the IDF MC convened its second annual bioethics conference, “The Oath and Accountability,” in July 2025. This event followed the inaugural conference held in June 2024, marking a sustained commitment to ethical discourse even amidst an active, broadening, multi-front war [11]. This article outlines the structure, methodology, and key thematic insights arising from the conference, arguing that the preservation of a robust ethical compass is essential for maintaining professional identity and effectiveness in an era of persistent conflict.

## Background: the ethical frameworks of the IDF MC

To understand the significance of the conference discussions, it is necessary to contextualize the ethical frameworks governing the IDF MC. The conduct of medical personnel is guided by a convergence of three distinct ethical codes, which occasionally create tension. The foundational layer is “The Spirit of the IDF” (“Ruah Tzahal”, see *Text Box 1*) [12], first composed in 1994, which serves as the general ethical code for all Israeli soldiers. It outlines the core values, moral standards, and conduct expected of every serviceman and woman, regardless of their specific role.



This general military code is complemented by the “Hebrew Physician’s Oath” [13]. Formulated by Prof. Lipman-Heilprin in 1952, this civilian oath stands as the standard for medical practitioners in Israel. Rooted in broad medical tradition, it emphasizes the intellectual and moral responsibilities of the physician, urging practitioners to “understand the patient’s soul” and restore their spirit through wisdom and compassion, focusing heavily on the character of the physician and their duty to the community. See *Text Box 2*.



However, the unique exigencies of military medicine are addressed by the “IDF Medical Corps Oath”, see *Text Box 3* [14]. Written by the lyricist Haim Hefer and introduced in the 1960s, this oath is distinct to the Corps. Unlike the civilian oath, it explicitly addresses the stark realities of the battlefield. It serves as a binding pledge for all medical personnel, not just physicians, but also paramedics, nurses, and combat medics. It commits personnel to “extend a helping hand. to friend or foe” and contains the uncompromising command to “never desert the wounded in the battlefield”.



The interplay between these codes, specifically the tension between the universal humanistic duties of the physician represented by the Hebrew Oath, and the particularistic, operational duties of the soldier represented by the Corps Oath, formed the intellectual foundation of the conference.

## Conference methodology and structure

The conference was hosted at the Shalom Hartman Institute in Jerusalem, a venue selected to facilitate deep, value-based inquiry. The event brought together approximately 200 military healthcare leaders, representing a deliberate and wide-ranging cross section of the Medical Corps. To ensure a holistic view of the medical system, participants included senior command officers, battalion-level physicians, general practitioners, dentists, nurses, mental health officers, physiotherapists, pharmacists, medics, and legal advisors. Notably, members of the institutional Ethics Committee were also in attendance to provide valuable guidance.

The conference structure was designed to move from high level conceptual frameworks to concrete, bottom-up application through a continuous flow of three primary segments. The day began with plenary sessions intended to establish a shared vocabulary. Rabbi Dr. Daniel Hartman, President of the Shalom Hartman Institute, offered a historical and social perspective on the ethical foundations of military medical service. This was followed by a lecture from the IDF Military Advocate General, who addressed the legal frameworks governing medical conduct in the battlefield and the intersection of law and ethics under combat conditions.

Following the plenary sessions, participants engaged in reflective workshops. Divided into small groups, they critically analyzed the IDF MC Oath, deconstructing its text and comparing it with the “Spirit of the IDF” [12] and the civilian “Hebrew Physician’s Oath” [13]. The day concluded with a session dedicated to case analysis, focusing on real-life ethical dilemmas arising from the current war presented by field personnel. A key methodological element was the facilitation of these small group discussions by moderators from the Shalom Hartman Institute, in collaboration with members of the IDF MC’s Ethics Committee. The explicit objective was to avoid functioning as a disciplinary hearing or rushing toward binary solutions. Instead, the facilitators aimed to encourage reflection, foster cross-disciplinary dialogue, and strengthen ethical critical thinking, providing a “safe space” for processing the emotional and moral residue of the war.

## Results: Thematic analysis of the medical corps oath

The examination of the Medical Corps Oath [14] yielded several distinct themes regarding professional identity and wartime accountability. A distinguishing feature of the Oath is its universality; unlike civilian medical oaths which apply primarily to doctors, the MC Oath binds every soldier in the Corps. Discussions highlighted the significance of this inclusivity. In the military setting, young, conscripted medics, who undergo only months of training, are often the first responders in extreme situations. The conference affirmed that these young soldiers are held to the same high moral standards demanded by the oath as senior physicians, requiring a robust ethical education that makes the oath accessible and relevant to them.

Furthermore, the Oath’s command to treat “any fellow man,” whether “friend or foe”, emerged as a focal point of intense discussion. While this principle is standard in medical ethics, its application in the context of the October 7th atrocities and the subsequent war, posed significant emotional challenges for caregivers. Participants discussed the “dual identity” of being both a soldier defending their country and a caregiver committed to universal humanity. The consensus that was reached reinforced the Oath as an essential barrier against the dehumanization of the enemy, preserving the medical professional’s integrity even in the face of “atrocities inflicted on Israelis”.

Finally, the clause “to never desert the wounded in the battlefield” [10] was subject to rigorous debate. Participants contrasted this with the civilian Hebrew Physician’s Oath, which focuses on the patient’s soul and spirit, whereas the military oath emphasizes physical solidarity and rescue. The discussion revealed a tension between this absolute value and the realities of combat warfare in austere environments, where triage protocols or threats to the safety of the medical force might necessitate leaving the wounded behind temporarily. The dialogue clarified that this clause represents a “Commandment of sacrifice”, blurring the lines between a medical duty and a commander’s responsibility.

### Operational dilemmas: insights from the field

The “bottom-up” small group discussions provided a vital window into the intricate ethical landscape of the “Swords of Iron” war, illustrating that such dilemmas are not rare contingencies but daily operational realities. For this segment of the conference, a selection of real-life cases encountered by IDF Medical Corps personnel throughout the conflict was compiled in advance. Each case was presented to a discussion group of approximately 15 participants by the individuals themselves (ranging from senior officers to junior practitioners) who had personally grappled with these dilemmas in the field.

The primary objective of these sessions was to deconstruct each case, surface its inherent ethical complexities, and explore potential resolutions, whether as a concrete response to the specific event or as a “prototype” for managing analogous situations in the future. To ensure a completely open, candid, and authentic exchange within a “safe space,” these discussions were not documented, and no formal minutes or institutional records were maintained. Consequently, while the sessions did not aim to produce formal systemic conclusions, they allowed both presenters and participants to derive significant personal insights and professional frameworks for their continued service.

The following three cases illustrate the broad spectrum of these challenges:

The first case highlighted the tension between empathy and operational necessity. A frontline medical team was faced with determining the fitness-for-duty of a fighter pilot holding a crucial combat role. While the pilot was suffering from a medical condition that warranted grounding, the medical officers faced immense pressure from line commanders to reinstate him immediately due to urgent, high stakes operational needs. This case highlighted the burden on the medical officer to serve as a gatekeeper, bringing into sharp focus the conflict between the medical obligation to the patient’s individual health and the military obligation to the collective security mission.

A second case, originating from routine wartime care, addressed the issue of patient autonomy within a uniformed system. A soldier suffering from a severe illness refused the recommended life-saving treatment. This scenario sparked a debate regarding the limits of patient autonomy within a rigid military hierarchy especially during wartime. Participants explored how the “Patients’ Rights Law” [15] interacts with military orders, and the role of the medical officer in protecting the soldier’s right to choose - even when that choice conflicts with the system’s expectations or military readiness.

A third case offered a unique perspective on “battlefield” ethics, drawn from the Home Front Command experience during the war campaign against Iran and its long-range missiles waged against Israel. During a rescue and evacuation operation at a missile impact site in a dense urban area, the medical force came under renewed fire. The force commander faced an instantaneous dilemma: continue the lifesaving treatment and evacuation of civilian casualties, thereby exposing the medical team to imminent danger, or prioritize “force protection” and seek cover. This scenario sparked an intense discussion regarding the scope of the “Commandment of sacrifice” and the balance between the safety of the caregiver and the duty to the patient in an asymmetric urban combat zone.

These examples highlighted that ethical dilemmas and value clashes encompass both extreme wartime conditions and everyday military medicine.

## Discussion

Holding a bioethics conference during an ongoing, multi-front war might seem, to an outside observer, as an untimely distraction. However, the feedback from this conference suggests the opposite. Participants noted that the act of pausing operational routine to engage in ethical reflection was an “act of strength” rather than aloofness. The conference reinforced the idea that ethical dialogue within the military medical community is vital for operational resilience. Confronting dilemmas head-on, rather than avoiding them, fosters unity, sharpens judgment, and ultimately elevates the standard of care provided to all individuals, including enemy combatants, civilians, and fellow soldiers. This aligns with broader military ethics literature, which suggests that maintaining moral clarity is essential for preventing burnout and moral injury in persistent conflict [8]. Theoretical frameworks further support the criticality of such engagement, identifying ‘openness to discuss ethical issues’ as a distinct cultural dimension that actively mitigates unethical behavior.

The conference discussions resonated with the work of bioethicists such as the late George Annas [16] and the late Edmund Pellegrino [17], particularly regarding the “dual loyalty” conflict, as further explored by Gross [18], van Es [19], Eze, Innoeze [20], and Brockie [21]. The question of whether one is a “physician first” or “soldier first” was a recurring motif. The conference reinforced the view that the IDF MC’s Oath serves as a compass, ensuring that medical ethics remain consistent. By reaffirming their commitment to the Oath, participants validated their professional identity, ensuring that the “soldier” aspect does not subsume the “healer” aspect.

Moreover, the conference underscored that the role of the IDF MC officer extends beyond clinical treatment. Commanders in the Medical Corps are embedded within combat units and serve as advisors to line commanders. This advisory function relies heavily on what Zheng et al. term ‘ethical voice’ – the willingness to speak up and influence decision-making based on deeply internalized values [22]. By cultivating a strong ‘integrity identity’ [22], medical officers are better equipped to advise commanders on complex decisions involving human life, evacuation priorities, and the treatment of non-combatants or enemies. This transforms the medical officer from a technical provider into a strategic advisor on values and morality in combat.

## Conclusion and future directions

The “Oath and Accountability” conference concluded with a commitment to embed values-based dialogue into routine practice. It catalysed a broader commitment to integrating ethical education and considerations into operational life and decision-making at all levels, with many commanders planning to initiate similar unit-level discussions, creating additional opportunities for ethical analysis and reflection closer to the field.

Following the gathering, several directions are currently being explored to preserve and formalize this engagement. These include the formulation of a formal Medical Corps code of ethics to bridge the gap between general military values and specific clinical duties, as well as the development of structured ethics education programs at the military medical academy. Additionally, the Corps is looking to establish collaborations with foreign military medical systems to exchange best practices in wartime ethics.

Ultimately, the Second Annual Bioethics Conference demonstrated that for the IDF Medical Corps, the Oath is not a ceremonial relic of the past, but a living principle guiding action in the most challenging of times. It is precisely through this willingness to confront ethical dilemmas head-on that the Corps reaffirms its enduring commitment to the principle that even amidst the chaos of war, medicine remains a deeply humanistic endeavor, guided by an unwavering oath.

## Data Availability

No datasets were generated or analysed during the current study.

## Abbreviations

IDF: Israel Defense Forces
MC: Medical Corps
